# *BMC Health Services Research* reviewer acknowledgement 2015

**DOI:** 10.1186/s12913-016-1311-z

**Published:** 2016-02-17

**Authors:** Christopher Morrey

**Affiliations:** BioMed Central, Floor 6, 236 Gray’s Inn Road, London, WC1X 8HB UK

## Abstract

The editors of *BMC Health Services Research* would like to thank all our reviewers who have contributed to the journal in Volume 15 (2015).

**Perri 6**

UK

**Ignacio Abaitua**

Spain

**Wendel Abel**

Jamaica

**Mubarek Abera**

Ethiopia

**Gilbert Abotisem Abiiro**

Ghana

**Emmanuel Aboagye**

Sweden

**Aaron Abuosi**

Ghana

**Timothy Abuya**

Kenya

**Dionisio Acosta**

UK

**Louise Ada**

Australia

**Susie Adams**

USA

**Adedeji Adekanye**

Nigeria

**Rajatashuvra Adhikary**

USA

**Adedayo Adio**

Nigeria

**Raphael Adobor**

Norway

**Martin Adokiya**

Ghana

**Gabriel Agboado**

UK

**Asgar Aghaei Hashjin**

Netherlands

**Irene Akua Agyepong**

Ghana

**Kees Ahaus**

Netherlands

**Riris Andono Ahmad**

Indonesia

**Mohamed Azmi Ahmad Hassali**

Malaysia

**Saifuddin Ahmed**

USA

**Ajibade Aibinu**

Australia

**Moses Aikins**

Ghana

**Mara Airoldi**

UK

**Budi Aji**

Indonesia

**Manabu Akazawa**

Japan

**Aslihan Akpinar**

Turkey

**Fadi Al Zoubi**

Canada

**Olufunke Alaba**

South Africa

**Fares Alahdab**

USA

**Sinaa Alaqeel**

Saudi Arabia

**Sandra Alba**

Netherlands

**Jennifer Albrecht**

USA

**Maria Alcaide**

USA

**Yibeltal Alemayehu**

USA

**Moazzam Ali**

Switzerland

**Bachti Alisjahbana**

Indonesia

**Jacqui Allen**

Australia

**Steve Allsop**

Australia

**Olakunle Alonge**

USA

**Keri Althoff**

USA

**Soumya Alva**

USA

**Dale Alverson**

USA

**Joana Alves**

Portugal

**Sarah Amador**

UK

**Charles Ameh**

UK

**Rivet Amico**

USA

**Eugenia Amporfu**

Ghana

**Ji-Young An**

South Korea

**Raghupathy Anchala**

India

**Elizabeth Andersen**

Canada

**Lena Andersson**

Sweden

**Aris Angelis**

UK

**Ashley Anker**

USA

**Fernando Antonanzas**

Spain

**Philippe Apparicio**

Canada

**Norm Archer**

Canada

**Virginia Arechavala-Gomeza**

Spain

**Olatunde Aremu**

UK

**Heike Argstatter**

Germany

**Patrizio Armeni**

Italy

**Nigel Armfield**

Australia

**Isabelle Arnet**

Switzerland

**I Wayan Gede Artawan Eka Putra**

Indonesia

**Eric Arthur**

Ghana

**Meghana Aruru**

USA

**Keiko Asao**

USA

**Toni Ashton**

New Zealand

**Sandy Askew**

USA

**Bouchra Assarag**

Morocco

**Foroozan Atashzadeh**

Iran

**Kostas Athanasakis**

Greece

**Helen Atherton**

UK

**Roger Ayimbillah Atinga**

Ghana

**Katherine Atkins**

UK

**Anita Atwal**

UK

**Christian Auer**

Switzerland

**Hanna Augustsson**

Sweden

**Isabelle Aujoulat**

Belgium

**Richard Ayah**

Kenya

**Tadesse Ayele**

Ethiopia

**Kieran Ayling**

UK

**Darshini Ayton**

Australia

**Lilian Azzopardi**

Malta

**Ca Baan**

Netherlands

**Giridhara R Babu**

India

**Omar Badawi**

USA

**Sung-Heui Bae**

USA

**Billy Bai**

USA

**Charles Bailey**

USA

**Ross Bailie**

Australia

**Joshua Baker**

USA

**Judith Baker**

USA

**Hector Balcazar**

USA

**Cassia Baldini Soares**

Brazil

**Francesco Balducci**

Italy

**Jane Banaszak-Holl**

USA

**David Banham**

Australia

**Jonathan Banks**

UK

**Finian Bannon**

UK

**Vishal Bansal**

India

**Sheheryar Banuri**

USA

**Yan-Ping Bao**

China

**Hamid Reza Baradaran**

Iran

**Pamela Barbadoro**

Italy

**Carolina Barbosa**

USA

**Lesley Barclay**

Australia

**Serena Barello**

Italy

**Morris Barer**

Canada

**Fiona Barker**

UK

**Paul Barnett**

USA

**Barbara Barrett**

UK

**Bruce Barron**

USA

**Régis Barros**

Brazil

**Kate Bartlem**

Australia

**Pelham Barton**

UK

**Melanie Barwick**

Canada

**Robert Basaza**

Uganda

**Philip Batterham**

Australia

**Sebastian Bauhoff**

USA

**Cynthia Baur**

USA

**Steffen Bayer**

Singapore

**Cynthia Bearer**

USA

**Angela Beck**

USA

**Anders Beckman**

Sweden

**Rinad Beidas**

USA

**Kimon Bekelis**

USA

**Simon Bell**

Australia

**David Bell**

UK

**Rhonda Belue**

USA

**Miriam Bender**

USA

**Khalid Benkhadra**

USA

**Tim Benson**

UK

**Petra Benzinger**

Germany

**David Beran**

Switzerland

**Paola Berchialla**

Italy

**Carl Berdahl**

USA

**Katherine Berg**

Canada

**Karin Berger**

Germany

**Anne-Marie Bergh**

South Africa

**Johannes Berkhof**

Netherlands

**Lars Bernfort**

Sweden

**Marian Betz**

USA

**Linda Beuscher**

USA

**Jennifer Bevan**

USA

**Sunil Bhandari**

UK

**Ramjee Bhandari**

UK

**Shafi Bhuiyan**

Canada

**Hazel Biggs**

UK

**Antonia Biggs**

USA

**Jean Joel Bigna**

Cameroon

**Catherine Binkley**

USA

**Colin Binns**

Australia

**Victoria Bird**

UK

**Alexander Bischoff**

Switzerland

**Marie Bismark**

New Zealand

**Anne Mette Bjerkan**

Norway

**Kirsten Black**

Australia

**Daniel Blackburn**

UK

**Regis Blais**

Canada

**Karl Blanchet**

France

**Marc-Andre Blanchette**

Canada

**Christopher Blanchette**

USA

**Ilse Blignault**

Australia

**Evan Bloch**

USA

**Eva Blozik**

Germany

**Said Bodur**

Turkey

**Dorthe Boe Danbjørg**

Denmark

**Benjamin Boerebach**

Netherlands

**Ties Boerma**

Switzerland

**Stefan Boes**

Switzerland

**Thomas Böhler**

Germany

**Laura Bojke**

UK

**Brandon Bookstaver**

USA

**Cecile Rl Boot**

Netherlands

**Jonathan Boote**

UK

**Josephine Borghi**

UK

**Judith Borja**

Philippines

**Tracey Borland**

Canada

**Elin Børøsund**

Norway

**Iris Borowy**

Germany

**Rohan Borschmann**

UK

**Renata Bortolus**

Italy

**Ann Borzecki**

USA

**Graham Bothamley**

UK

**Markella Boudioni**

UK

**Mary Boulton**

UK

**Alida Bouris**

USA

**Chris Bourne**

Australia

**Tom Boyles**

South Africa

**Richard Bradley**

USA

**Anne-Marie Brady**

Ireland

**Wornei Braga**

Brazil

**Liz Brester**

UK

**Cédric Bretonnière**

France

**Timothy Brewer**

USA

**Abenaa Brewster**

USA

**Jane Brice**

USA

**John F P Bridges**

USA

**William Brieger**

USA

**Jo-Anne Brien**

Australia

**Bianca Brijnath**

Australia

**Katie Bristow**

UK

**Thomas Britt**

Vanuatu

**Mark Brittan**

USA

**Jonathan Broadbent**

New Zealand

**Edward Broughton**

USA

**Brandon Brown**

USA

**Tim Bruckner**

USA

**Suanna Bruinooge**

USA

**Francesca Brundisini**

Canada

**Nele Brusselaers**

Sweden

**Denise Bryant-Lukosius**

Canada

**Patrick Brzoska**

Germany

**Luca Buccoliero**

Italy

**Andrea Bullock**

USA

**Eduardo Bunge**

USA

**Alicia Bunger**

USA

**Rita Burke**

USA

**Sheretta Butler-Barnes**

USA

**Peter Byass**

Sweden

**Gobnait Byrne**

Ireland

**Jens Byskov**

Zambia

**Chiara Cadeddu**

Italy

**Sean Cahill**

USA

**Amaia Calderón-Larrañaga**

Spain

**Peter Callery**

UK

**Francesca Calo**

UK

**Claire Cameron**

New Zealand

**John Campbell**

UK

**Robert Campbell**

USA

**Carlos Campillo-Artero**

Spain

**Kenneth Candido**

USA

**Robyn Cant**

Australia

**Michael Cantor**

USA

**Michael Cantor**

USA

**Cristina Caperchione**

Canada

**Alejandra Caqueo-Urizar**

Chile

**Esteban Cardemil**

USA

**Victoria Cardenas**

Australia

**David Carlbom**

USA

**Siw Carlfjord**

Sweden

**Jan D Carline**

USA

**Catherine Carr**

UK

**Henry Carretta**

USA

**Ken Carswell**

UK

**Nancy Carter**

Canada

**Anna Cristina Carvalho**

Italy

**Anne-Nicole Casey**

Australia

**Patty Castelli**

USA

**Víctor H. Castillo**

Mexico

**Jessica Castner**

USA

**Enrique Castro-Sánchez**

UK

**Christine Catling**

Australia

**Gillian Caughey**

Australia

**Giovanni Luca Ceresoli**

Italy

**Christa Cerra**

USA

**Monique Chaaya**

Lebanon

**David Chambers**

USA

**Chien-Lung Chan**

Taiwan

**Alvin Chandra**

USA

**Hsiao-Ting Chang**

Taiwan

**Bei-Hung Chang**

USA

**Evelyn Chang**

USA

**Jianqian Chao**

China

**Arjun Chatterjee**

USA

**Kath Checkland**

UK

**Jack Chen**

Australia

**Wen Chen**

China

**Elaine Chen**

USA

**Zhuo Chen**

USA

**Yingyao Chen**

China

**Jean-Francois Chenot**

Germany

**Nicola Cherry**

Canada

**Helen Cheyne**

UK

**Mary Chiarella**

Australia

**Carlos Chiatti**

Italy

**Gabriel Chodick**

Israel

**Mona Choi**

South Korea

**Virasakdi Chongsuvivatwong**

Thailand

**Angela Chow**

Singapore

**Askar Chukmaitov**

USA

**Deborah Chyun**

USA

**Fiona Cianci**

Ireland

**Oriana Ciani**

UK

**Julie Cichero**

Australia

**Nesrin Çilingiroglu**

Turkey

**Patricia Clark**

Mexico

**Antonio Clavenna**

Italy

**Ciara Close**

Ireland

**Sean Clouston**

USA

**Noelle Cocoros**

USA

**Arnon Dov Cohen**

Israel

**Helen Coleman**

UK

**Anna Coleman**

UK

**Karen Collins**

UK

**Mercedes Colomar**

Uruguay

**Heather Colquhoun**

Canada

**Christopher Colvin**

South Africa

**Dagna Constenla**

USA

**Lesong Conteh**

UK

**Kieran Cooley**

Canada

**Eleanor Cornford**

UK

**Massimo Costantini**

Italy

**Francesc Cots**

Spain

**Cheryl Cott**

Canada

**Neil Cottrell**

Australia

**Susan Cottrell**

UK

**Jennifer Couturier**

Canada

**Yves Couturier**

Canada

**Sophia Couzos**

Australia

**Consuelo Coy**

Australia

**Deborah Crabbe**

USA

**Louise Craig**

Australia

**Gregory Crawford**

Australia

**Andreea Creanga**

USA

**Kathrin Cresswell**

UK

**Bart Criel**

Belgium

**Julio Croda**

Brazil

**Neeltje Crombag**

Netherlands

**Rose Crowley**

UK

**Clare Crowley**

UK

**Frances Cunningham**

Australia

**Maria Paula Curado**

France

**Jane Currie**

Australia

**Marek Cybulski**

Poland

**Ann Dadich**

Australia

**Joanne Daggy**

USA

**Rada Dagher**

USA

**Sherry Dahlke**

Canada

**Baozhen Dai**

China

**Helen Dakin**

UK

**Maxwell Ayindenaba Dalaba**

Ghana

**Maurice Dalton**

USA

**Gianfranco Damiani**

Italy

**Konstantinos Danas**

UK

**Elvira D'Andrea**

Italy

**Carolina Danovaro-Holliday**

USA

**Andriy Danyliv**

Ireland

**Carl D'Arcy**

Canada

**Mrinalini Das**

India

**Umakant Dash**

India

**Adam Davey**

USA

**Amy Davidoff**

USA

**Amy L. Davidow**

USA

**Amy Davidow**

USA

**Thomas Davidson**

Sweden

**Barbara Davies**

Canada

**Carol Davy**

Australia

**Karen Day New**

Zealand

**Wout De Boer**

Switzerland

**Margaretha Christine De Bruijne**

Netherlands

**Ayesha De Costa**

Sweden

**Thyra De Jongh**

Netherlands

**Simon De Lusignan**

UK

**Jeroen De Mast**

Netherlands

**Paula De Toledo**

Spain

**Anniza De Villiers**

South Africa

**Chiara De Waure**

Italy

**G.Ardine De Wit**

Netherlands

**Lisa Decamp**

USA

**Matthew Decamp**

USA

**Irene R Degano**

Spain

**Howard Degenholtz**

USA

**Christian Dehlendorff**

Denmark

**Mary E Deily**

USA

**Paul Delamater**

USA

**Derek Delia**

USA

**Dianne Delva**

Canada

**Thérèse Delvaux**

Benin

**Sarang Deo**

India

**Maral Dersarkissian**

USA

**Laura Di Giorgio**

USA

**Mirko Di Rosa**

Italy

**Vakaramoko Diaby**

USA

**Karam Diaby**

USA

**Hassan H. Dib**

Canada

**Kristen Didonato**

USA

**Karin Dieperink**

Denmark

**Jens Dietrichson**

Sweden

**Vance Dietz**

USA

**Janet Diffin**

UK

**Helen Dimaras**

Canada

**Michaela Dinan**

USA

**Geert-Jan Dinant**

Netherlands

**Birthe Dinesen**

Denmark

**Yan Ding**

Germany

**Jenna Dixon**

Canada

**Josephine Dixon**

UK

**Fahdi Dkhimi**

Belgium

**Mai Do**

USA

**Henry Doctor**

Nigeria

**Maman Joyce Dogba**

Canada

**Stephen Doherty**

Australia

**Sally Doherty**

Ireland

**Siobhan Dolan**

USA

**Maria Donald**

Australia

**Faith Donald**

Canada

**Andrea Donatini**

Italy

**France Donnay**

USA

**Michael Donnelly**

UK

**Lena Dorin**

Germany

**William Doucette**

USA

**Athanassios Douzenis**

Greece

**Julia Downing**

Uganda

**Colleen Doyle**

Australia

**Frank Doyle**

Ireland

**Ann Dozier**

USA

**Paul Drain**

USA

**Albert Dreijer**

Netherlands

**David Dror**

India

**Joseph Drozda**

USA

**Thomas Druetz**

Canada

**Frances Drummond**

Ireland

**Nick Drydakis**

UK

**Stephen Duckett**

Canada

**Danny Duke**

USA

**Paul Duncan**

USA

**Angela Durey**

Australia

**Nilanjana Dwibedi**

USA

**Dagrunn Nåden Dyrstad**

Norway

**Krysia Dziedzic**

UK

**Bassey Ebenso**

UK

**Dale Edgar**

Australia

**Richard Edlin**

New Zealand

**Louisa Edwards**

UK

**Paul Effler**

Australia

**Monica Eftedal**

Norway

**Mary Egan**

Canada

**Carolyn Ehrlich**

Australia

**Adam Ehrlich**

USA

**Ricardo Eiraldi**

USA

**Andreia Eiras**

Portugal

**Sheri Eisert**

USA

**Björn Ekman**

Sweden

**Miriam Elfström**

Sweden

**Moriah Ellen**

Israel

**Michael Elliott**

USA

**David Ellwood**

Australia

**Anashua Rani Elwy**

USA

**Obiageli Emelumadu**

Nigeria

**Lynne Emmerton**

Australia

**Tracy Epton**

UK

**Osaro Erhabor**

Nigeria

**Brigitte Essers**

Netherlands

**Adrian Esterman**

Australia

**Enyi Etiaba**

Nigeria

**Stefanie Ettelt**

UK

**Bill Evans**

Canada

**Roni Evans**

USA

**Charles Ezenduka**

Nigeria

**Nkoli Ezumah**

Nigeria

**Joseph Fadare**

Nigeria

**Jean-Benoit Falisse**

UK

**Victoria Fan**

USA

**Victoria Fan**

USA

**Pengqian Fang**

China

**Maria Pia Fantini**

Italy

**Wigdan Farah**

USA

**Rita Faria**

UK

**Robyn Fary**

Australia

**Mark Faul**

USA

**Edward Vincent S. Faustino**

USA

**Naomi Fearns**

UK

**Frank Federico**

USA

**Frank Feeley**

USA

**Paul Feldblum**

USA

**Zhanchun Feng**

China

**Laurie Anne Ferguson**

USA

**Yann Ferrand**

USA

**Alessandra Ferrario**

UK

**Marie Ferrua**

France

**Deirdre Fetherstonhaugh**

Australia

**Kathryn Field**

Australia

**Robert Field**

USA

**Jane Figuieredo**

USA

**Guido Filler**

Canada

**Joseph Fink**

USA

**Michael Fischer**

USA

**Andrea Fischl**

USA

**Ray Fitzpatrick**

UK

**Sarah Flanagan**

UK

**Steffen Flessa**

Germany

**Mirembe Florenc**

Uganda

**Carole Fogg**

UK

**Ronan Foley**

Ireland

**James Ford II**

USA

**Roberto Forero**

Australia

**Lynne Forrest**

UK

**Yvonne Forsell**

Sweden

**Della Forster**

Australia

**Vassilis Fragoulakis**

Greece

**Giuseppe Franchino**

Italy

**Matthew Franklin**

UK

**Shawn Fraser**

Canada

**Suzanne Fredericks**

Canada

**Gerardus Frederix**

Netherlands

**Simon French**

Canada

**Stanley Frencher Jr**

USA

**Atle Fretheim**

Norway

**Paula Frew**

USA

**Jeremy Friedman**

Canada

**Caren Frost**

USA

**Marie Fuglsang**

Denmark

**Jeffrey Fuller**

Australia

**Judith Fullerton**

USA

**Elena Fumagalli**

Switzerland

**Martha Funnell**

USA

**Jennifer Furin**

USA

**Josep Fuste**

Spain

**Roger Gadsby**

UK

**Joshua Gagne**

USA

**Caroline Gaither**

USA

**Petros Galanis**

Greece

**Elizabeth Galik**

USA

**Elvis Gama**

UK

**Dashiell Gantner**

Australia

**Jianmin Gao**

China

**Zan Gao**

USA

**Guodong Gao**

USA

**Anibal García-Sempere**

Spain

**Anne Helene Garde**

Denmark

**Pernilla Garmy**

Sweden

**Melissa Garrido**

USA

**Benjamin Gartrell**

USA

**Jennifer Garvin**

USA

**Etienne Gayat**

France

**Gebremedhin Berhe Gebregergs**

Ethiopia

**Hailemichael Gebreselaissie**

Ethiopia

**Stacie Geller**

USA

**Paul Gemmel**

Belgium

**Johnson George**

Australia

**Caitlin Gerdts**

USA

**Chloeger Gervès-Pinquié**

France

**Johannes Geyer**

Germany

**Setareh Ghahari**

Canada

**Alessandro Ghidini**

USA

**Simone Ghislandi**

Italy

**Suparna Ghosh-Jerath**

India

**Francesco Gianfagna**

Italy

**Peter Gibbs**

Australia

**Simone Gibson**

Australia

**Wendy Gifford**

Canada

**Kenneth Gill**

USA

**Christopher Gill**

USA

**Brigid Gillespie**

Australia

**James Gillespie**

Australia

**Mattia Gilmartin**

USA

**Ronald Gimbel**

USA

**Liane Ginsburg**

Canada

**Carlo Bruno Giorda**

Italy

**Jessica Gipson**

USA

**Edmond Girasek**

Hungary

**Jody Hoffer Gittell**

USA

**Andrea Giusti**

Italy

**Richard Glazier**

Canada

**Valeria Glorioso**

Italy

**Dianne Godkin**

Canada

**Brian Godman**

Sweden

**Michael Goepfert**

UK

**Rosa Gofin**

USA

**Rachel Gold**

USA

**Peter Goldblatt**

UK

**Margalit Goldfracht**

Israel

**David Goldsbury**

Australia

**Jorge Fabricio González**

Spain

**Victoria Goodwin**

UK

**Nicholas Goodwin**

UK

**Susan Goold**

USA

**Sophie Gorgemans**

Spain

**Martin Gorsky**

UK

**Elena Gospodarevskaya**

Australia

**Neela Goswami**

USA

**George Gotsadze**

Georgia

**George Gourzoulidis**

Greece

**Indiran Govender**

South Africa

**Abhinav Goyal**

USA

**Pedro Gozalo**

USA

**Christopher Graber**

USA

**Markus Grabka**

Germany

**Sandra Grace**

Australia

**Ilana Graetz**

USA

**Chris Graham**

UK

**Aileen Grant**

UK

**Robert Grant**

UK

**Garry Gray**

Canada

**Samantha Gray**

Canada

**Andrew Lofts Gray**

South Africa

**Christen Gray**

UK

**Michael Greco**

Australia

**Nancy Green**

USA

**Geva Greenfield**

UK

**Jeff Greenwald**

USA

**David Grembowski**

USA

**Sverre Grepperud**

Norway

**Rosane Griep**

Brazil

**Ulla Griffiths**

UK

**Erik Groessl**

USA

**Vigdis Abrahamsen Grøndahl**

Norway

**Amy Grove**

UK

**Andrea Gruneir**

Canada

**Yuanyuan Gu**

Australia

**Maria Rosaria Gualano**

Italy

**Mara Guerreiro**

UK

**Erick Guerrero**

USA

**Nalika Gunawardena**

Spain

**Shailvi Gupta**

USA

**Bruce Gurd**

Australia

**Sebastian Gurtner**

Germany

**Kilian Gust**

Germany

**Rikke Gut**

Denmark

**Inaki Gutierrez**

Spain

**Ines Guttmann-Bauman**

USA

**Margaret Gyapong**

Ghana

**Ju-Young Ha South**

Korea

**Jeannie Haggerty**

Canada

**Jennifer Hahn-Holbrook**

USA

**Cherona Hajewski**

USA

**Mythri Halappa**

India

**Nafisa Halim**

USA

**Rasheeda Hall**

USA

**Heidemarie Haller**

Germany

**Francoise Hamers**

France

**Antje Hammer**

Germany

**Marianne Hanning**

Sweden

**Kristian Hansen**

UK

**Claudia Hanson**

Sweden

**Jing Hao**

USA

**Danielle Harari**

UK

**Samantha Harden**

USA

**Christopher Harle**

USA

**David Harley**

Australia

**Mark Harris**

Australia

**Simon Harrison**

Australia

**Christine Hartmann**

USA

**Carol Harvey**

Australia

**Gill Harvey**

Australia

**Leslie Hasche**

USA

**Arvid Steinar Haugen**

Norway

**Mona Haugum**

Norway

**Steven Hawken**

Canada

**Alan Haycox**

UK

**Catherine Hayes**

Ireland

**Philip Haywood**

Australia

**Bethany L Hedt-Gauthier**

USA

**Ramzi Helewa**

Canada

**Karla Hemesath**

USA

**Julie Henderson**

Australia

**Joanna Henderson**

Canada

**Joyce Hendricks**

Australia

**Jane Hendy**

UK

**Kevin Henry**

USA

**Abraham Herbst**

South Africa

**Kurt Hersberger**

Switzerland

**Christo Heunis**

South Africa

**Suzanne Heurtin-Roberts**

USA

**Patricia Heyn**

USA

**Madelyn Hsiao-Rei Hicks**

USA

**Niall Higgins**

Australia

**Anne-Marie Hill**

Australia

**Suzanne Hill**

Australia

**Peter Hill**

Australia

**Jennie Hill**

USA

**Janet Hiller**

Australia

**Susan Hillier**

Australia

**Shela Hirani**

Pakistan

**Lisa Hirschhorn**

USA

**Vivian Ho**

USA

**Ha Hoang**

Australia

**Matthias Hoben**

Canada

**Matthias Hochadel**

Germany

**Harry Hochheiser**

USA

**Stephen Hodgins**

USA

**Gareth Hollands**

UK

**Sandra Hollinghurst**

UK

**Samantha Hollingworth**

Australia

**Christine Holmberg**

Germany

**Maurits Hoogerwerf**

Netherlands

**Ann Hopton**

UK

**Frances Horgan**

Ireland

**Simon Horsburgh**

New Zealand

**Krishna Hort**

Australia

**David Hotchkiss**

USA

**David Howard**

USA

**Heather Howard**

USA

**Fei-Yuan Hsiao**

Taiwan

**William Hsiao**

USA

**Chin-Ying Hsu**

Singapore

**Clarissa Hsu**

USA

**I-Ping Hsueh**

Taiwan

**Shanlian Hu**

China

**Zhengxing Huang**

China

**Yu-Tung Huang**

Taiwan

**Heather Huang**

USA

**Rebecca Hubbard**

USA

**Hervé Hubert**

France

**Peter Huckfeldt**

USA

**Claudia Huebner**

Germany

**Marco Huesch**

USA

**Balthasar L. Hug**

Switzerland

**Robbert Huijsman**

Netherlands

**Heather Hume**

Canada

**Marjan Hummel**

Netherlands

**Niamh Humphries**

Ireland

**Vania Hungria**

Brazil

**Matthew Hunt**

Canada

**John Huppertz**

USA

**Anna-Karin Hurtig**

Sweden

**Mehwish Hussain**

Pakistan

**Denise Hynes**

USA

**Ogochukwu Ibe**

Nigeria

**Hyacinth Ichoku**

Nigeria

**Ayumi Igarashi**

Japan

**Chukwunonso Iheoma**

Nigeria

**Petrida Ijumba**

Rwanda

**Miyuki Imanishi**

Japan

**Maria Inacio**

Australia

**Cesar Infante**

Mexico

**Tor Ingebrigtsen**

Norway

**Richard Ingram**

USA

**Anthea Innes**

UK

**Muhammad Zahid Iqbal**

Malaysia

**Taya Irizarry**

USA

**Wanrudee Isaranuwatchai**

Canada

**Mohammad Ismail**

Pakistan

**Michele Issel**

USA

**Laurel Issen**

UK

**Salvatore Italia**

Germany

**Hiroto Ito**

Japan

**Valentina Cabral Iversen**

Norway

**Hilde Hestad Iversen**

Norway

**Liisa Jaakkimainen**

Canada

**Ayham Jaaron**

Palestine

**Cynthia Jacelon**

USA

**Susan Jack**

Canada

**Claire Jackson**

Australia

**Debra Jackson**

USA

**Yves Jackson**

Switzerland

**Diana Jackson**

UK

**Richard Jacques**

UK

**Bawo James**

Nigeria

**Marilyn James**

UK

**Tania Janaudis-Ferreira**

Canada

**Anne-Sophie Jannot**

Switzerland

**Rick Jansen**

USA

**Lina Jaruseviciene**

Lithuania

**Geetha Jayaram**

USA

**Saroj Jayasinghe**

Sri Lanka

**Lianne Jeffs**

Canada

**Yun-Hee Jeon**

Australia

**Weiyan Jian**

China

**Conwoo Emmanuel Jo**

New Zealand

**William Joe**

India

**May-Lill Johansen**

Norway

**Ann John**

UK

**Benjamin Johns**

USA

**Mark R D Johnson**

UK

**Heather Johnson**

USA

**Timothy Johnson**

USA

**David Jolley**

UK

**Kate Jolly**

UK

**Claudio Jommi**

Italy

**Fiona Jones**

UK

**Carys Jones**

UK

**Ray Jones**

UK

**Lorelei Jones**

UK

**Andrew Jones**

UK

**Carolynn Jones**

USA

**Christopher Jones**

USA

**Christine Jorm**

Australia

**Chandni Joshi**

Australia

**Basant Joshi**

Nepal

**Ehsan Jozaghi**

Canada

**Christian Juhra**

Germany

**Elizabeth Juma**

Kenya

**Kyoungrae Jung**

USA

**Corrine Jurgens**

USA

**Prapan Jutavijitum**

Thailand

**Don Juzwishin**

Canada

**Ashley Kable**

Australia

**Chodziwadziwa Kabudula**

South Africa

**Karen Kaiser**

USA

**Daphne Kaitelidou**

Greece

**László Kalabay**

Hungary

**Sudhir Kale**

Australia

**Elsbeth Kalenderian**

USA

**Lisa Kalman**

USA

**Eva Kaltenthaler**

UK

**Vinay Kamat**

Canada

**Ngianga-Bakwin Kandala**

UK

**Rebecca Kandiyali**

UK

**Donghun Kang**

South Korea

**Srinivasan Kannan**

India

**Nathan Kapata**

Zambia

**Anu Kapilashrami**

UK

**Warren Kaplan**

USA

**Jim Kaput**

Switzerland

**Nilamadhab Kar**

UK

**Anup Karan**

India

**Prisca Kasonde**

Zambia

**Panagiotis Kasteridis**

UK

**Alan Katz**

Canada

**Aaron Katz**

USA

**Manmeet Kaur**

India

**Prabhdeep Kaur**

India

**Reem Kayyali**

UK

**Abby Kazley**

USA

**Patricia Kearney**

Ireland

**Guy Kegels**

Belgium

**Margaret Kelaher**

Australia

**Maureen Kelly**

Ireland

**James Kelly**

UK

**Paul Kennedy**

Australia

**Anne Kennedy**

UK

**Kate Kerber**

Canada

**Roselinde Kessels**

Belgium

**Andrew Kestler**

Canada

**Basile Keugoung**

USA

**Hanan Khalil**

Australia

**Anne Khasakhala**

Kenya

**Naresh Khatri**

USA

**Babak Khoshnood**

France

**Anne Killett**

UK

**Jonathan Kilworth**

UK

**Bum Jung Kim**

USA

**James Kimani**

Kenya

**Sharla King**

Canada

**Christopher King**

USA

**Leigh Kinsman**

Australia

**Eileen Kintner**

USA

**Aaron Kipp**

USA

**Laurence Kirmayer**

Canada

**Alison Kitson**

Australia

**Suzanne Kiwanuka**

Uganda

**Niels Kristian Kjaer**

Denmark

**Gustav Kjellsson**

Sweden

**Scott Klarenbach**

Canada

**Maria Kleinstäuber**

Germany

**Herschel Knapp**

USA

**Alec Knight**

UK

**Jennifer Knopp-Sihota**

Canada

**Sarah Knowles**

UK

**Steven Fredric Koch**

South Africa

**Leonardo Koeser**

UK

**Jillian Clare Kohler**

Canada

**Stefan Kohler**

Germany

**Brandon Kohrt**

USA

**Maryse Kok**

Netherlands

**Marin Kollef**

USA

**Anton Kolomeyer**

USA

**Antonis Kontemeniotis**

Cyprus

**Xander Koolman**

Netherlands

**Siran Koroukian**

USA

**Grigorios Kotronoulas**

UK

**Anita Kotwani**

India

**Stephen Kozey**

Canada

**Murray Krahn**

Canada

**Andrew Kramer**

USA

**Sara Kreindler**

Canada

**Hartmut Krentz**

Canada

**Anand Krishnan**

India

**Andrea Kriska**

USA

**Solvejg Kristensen**

Denmark

**Troels Kristensen**

Denmark

**Søren Rud Kristensen**

UK

**Jimmie Kristensson**

Sweden

**Maria Kristiansen**

Denmark

**Christian Kronborg**

Denmark

**Ian Kronish**

USA

**Janet Krska**

UK

**Ralf Krumkamp**

Germany

**Silvia Krumm**

Germany

**Clemens Scott Kruse**

USA

**Grace Marie Ku**

Philippines

**Ellen Kuhlmann**

Germany

**Asli Kulane**

Sweden

**Juhi Kumar**

USA

**Ramesh Kumar**

Pakistan

**Nilay Kumar**

USA

**Shubha Kumar**

USA

**Benjamin Kuntz**

Germany

**Tarja Kvist**

Finland

**Hye-Young Kwon**

South Korea

**Nicholas Kyei**

Ghana

**Lareina La Flair**

USA

**Ulrich Laaser**

Germany

**Tracey-Lea Laba**

Australia

**Dragana Lakic**

Serbia

**Christiaan Lako**

Netherlands

**Kamakshi Lakshminarayan**

USA

**Christophe Lalanne**

France

**Debbie Laliberte Rudman**

Canada

**Antonio M. Lallena**

Spain

**Jonathan Lamb**

UK

**Stephen Lambert**

Australia

**Sylvie Lambert**

Canada

**Jed Lampe**

USA

**Yulia Landa**

USA

**Filipa Landeiro**

UK

**Reid Landes**

USA

**Christine Landy**

Canada

**Gloria Langat**

UK

**Chunhuan Lao**

New Zealand

**Luís Lapão**

Portugal

**Hélène Laperrière**

Canada

**Charles Larson**

Canada

**Heidi Larson**

UK

**Gunter Laux**

Germany

**Michael Lauzardo**

USA

**Maggie Lawrence**

UK

**Michael Barton Laws**

USA

**Jeffrey Lazarus**

Denmark

**Gweneth Lazenby**

USA

**Amanda Leach**

Australia

**Emily Leckman-Westin**

USA

**Jinhyung Lee**

South Korea

**Shoou-Yih Daniel Lee**

USA

**Kelly Lee**

USA

**Federico Lega**

Italy

**David Legge**

Australia

**Sara Legrand**

USA

**Maarit Leinonen**

Finland

**Anja Leist**

Netherlands

**Margus Lember**

Estonia

**Terhi Lemetti**

Finland

**Lidwien Lemmens**

Netherlands

**Chryssoula Lemonidou**

Greece

**Natalie Leon**

South Africa

**Bethany Leonhardt**

USA

**Rein Lepnurm**

Canada

**Laurent Letrilliart**

France

**Ann Levin**

USA

**Carol Levin**

USA

**Robert Levine**

USA

**Michele Levinson**

Australia

**Terry Lewin**

Australia

**George Lewith**

UK

**Sonia Lewycka**

New Zealand

**Joel Lexchin**

Canada

**Guohong Li**

China

**Gui Li**

China

**Zhijian Li**

China

**Qinghua Li**

USA

**Xiaoyan Li**

USA

**Min Lian**

USA

**Lin Liao**

China

**Dianne Liebel**

USA

**Martina Lietz**

Germany

**Giorgio Liguori**

Italy

**Rosemary Lim**

UK

**Chung-Wei Christine Lin**

Australia

**Hualiang Lin**

China

**Linda Lindeke**

USA

**Björn Lindgren**

Sweden

**Helena Lindgren**

Sweden

**Martin Lindstrom**

Sweden

**Martin Lipsky**

USA

**Christopher Lis**

USA

**Alyson Littman**

USA

**Isabel Litwin-Davies**

Sweden

**Yan Liu**

Australia

**Chaojie Liu**

Australia

**Zhaorui Liu**

China

**Qin Liu**

China

**Lei Liu**

USA

**Peter Lloyd-Sherlock**

UK

**Malin Lohela-Karlsson**

Sweden

**Julia Lohmann**

Germany

**Qian Long**

China

**Andrew Long**

UK

**Sharon Long**

USA

**Willemijn Looman**

Netherlands

**Jesus Lopez-Alcalde**

Italy

**Daniel Lopez-Cevallos**

USA

**Paula Lorgelly**

Australia

**Mary Losch**

USA

**Hong Lu**

China

**Roberto Lucchini**

Italy

**Jeff Luck**

USA

**Dave Ludwick**

Canada

**Marjolein Lugtenberg**

Netherlands

**Huabin Luo**

USA

**May Nawal Lutfiyya**

USA

**Lu Luzheng**

China

**Renee Lyons**

Canada

**Steven M. Frank**

USA

**William M. Sappenfield**

USA

**Shuangge Ma**

USA

**John Maa**

USA

**Marion Maar**

Canada

**Douglas Macdonald**

USA

**Anne Macfarlane**

Ireland

**Adrian Mackenzie**

Canada

**David Maclaren**

Australia

**Catherine Macphail**

Australia

**Rosa Magallon-Botaya**

Spain

**Vuyo Magasana**

South Africa

**Pascal Magnussen**

Denmark

**Andrew Maiorana**

Australia

**Konstantinos Makaritsis**

Greece

**Flora Malamateniou**

Greece

**Chetna Malhotra**

Singapore

**Robert Malkin**

USA

**Ira Malmberg-Heimonen**

Norway

**Mary Malone**

UK

**Mats Malqvist**

Sweden

**Stephen Maluka**

Tanzania

**Toshie Manabe**

Japan

**Semira Manaseki-Holland**

UK

**Vinaya Manchaiah**

USA

**Kate Mandeville**

UK

**Christos Manes**

Greece

**Tanja Manser**

Switzerland

**Jill Manthorpe**

UK

**Jennifer Manuel**

USA

**Liang Mao**

USA

**Bruno Marchal**

Belgium

**Maureen Markle-Reid**

Canada

**Talar Markossian**

USA

**Guy Marks**

Australia

**Michael Marks**

UK

**Stefan Markun**

Switzerland

**Lena Marmstål Hammar**

Sweden

**Andréa Marques**

Portugal

**Elsa Marques**

UK

**Maurice Mars**

South Africa

**Paul Marschall**

Germany

**Lena Mårtensson**

Sweden

**Brook Martin**

USA

**Linda Martin**

Netherlands

**Graham Martin**

UK

**Jorge Eduardo Martinez Perez**

Spain

**Eliana Cristina Martins Miranda**

Brazil

**Christine Marton**

Canada

**Grant Martsolf**

USA

**Shiko Maruyama**

Australia

**Michael Marx**

Germany

**Bigdeli Maryam**

USA

**Erin Masterson**

USA

**Ceu Mateus**

Portugal

**Inke Mathauer**

Switzerland

**Maria Mathews**

Canada

**Joseph Matovu**

Uganda

**Judith Matthews**

USA

**Stephen Matthews**

USA

**Wendy Max**

USA

**Leonard Mboera**

Tanzania

**Marion McAllister**

UK

**Abigail McBirnie**

USA

**David McBride**

New Zealand

**Brian McCabe**

USA

**Geraldine McCarthy**

Ireland

**Mark McCarthy**

UK

**Dennis McCarty**

USA

**Margaret McConnell**

USA

**Jacqueline McCormack**

UK

**Noleen McCorry**

USA

**Michael McCrory**

USA

**Peter McCulloch**

UK

**Megan McCullough**

USA

**Vivia McCutcheon**

USA

**Reuben McDaniel**

USA

**Scott McDonald**

Netherlands

**Geoff McDonnell**

Australia

**Shelley-Ann McGee**

South Africa

**Sinead McGilloway**

Ireland

**Rosemary McGinnes**

Australia

**Emma McGinty**

USA

**Linda McGowan**

UK

**Matthew McGrail**

Australia

**Cynthia McGrath**

USA

**Cynthia McGrath**

USA

**Elizabeth McInnes**

Australia

**Brian McKenna**

Australia

**Pascal McKeown**

UK

**Britt McKinnon**

Canada

**Andrew McLachlan**

Australia

**Zoe McLaren**

USA

**Josephine McMurray**

Canada

**Harry McNaughton**

New Zealand

**Rebekah McNaughton**

UK

**Ian Stewart McRae**

Australia

**Graham Meadows**

Australia

**Ivlabehire Bertrand Meda**

Burkina Faso

**Araya Abrha Medhanyie**

Ethiopia

**Richard Meenan**

USA

**Ludwien Meeuwesen**

Netherlands

**Roshanak Mehdipanah**

Canada

**Amber Mehmood**

USA

**Nadja Meisterhans**

Germany

**Seema Meloni**

USA

**Ildefonso Mendez-Martinez**

Spain

**Shanthi Mendis**

Switzerland

**Matthew Menear**

Canada

**Aziz Merchant**

USA

**James Meredith**

UK

**Dominik Mertz**

Canada

**Addisu Mesfin**

USA

**Markus Messerli**

Switzerland

**Gabriele Messina**

Italy

**Scott Metcalfe**

New Zealand

**Gesine Meyer-Rath**

South Africa

**Natasha Michael**

Australia

**Nicos Middleton**

Cyprus

**Evelinn Mikkelsen**

Netherlands

**Terence Mills**

Australia

**Samuel Mills**

USA

**Silvano Mior**

Canada

**Claudia Miranda-Castillo**

Chile

**Reza Mirnezami**

UK

**Anne Mitchell**

Australia

**Carole Mitnick**

USA

**Terry Mizrahi**

USA

**Philipa Mladovsky**

UK

**Kristin Mmari**

USA

**George Mnatzaganian**

Australia

**Gita Mody**

USA

**Katia Mohindra**

Canada

**David Mohr**

USA

**Fiona Moir**

New Zealand

**Wieneke Mokkink**

Netherlands

**Karen Monsen**

USA

**Daniel Montanera**

USA

**Jesus Montero-Marin**

Spain

**Alicia Montgomerie**

Australia

**Alvaro Montiel-Jarquín**

Mexico

**Melinda Moore**

USA

**Elizabeth Moore**

USA

**Rafique Moosa**

South Africa

**Iain Moppett**

UK

**Alessandro Morandi**

Italy

**Gerardo Moreno**

USA

**Maria Morgan**

UK

**Kerri Morgan**

USA

**Kate Morris**

UK

**Maggie Mort**

UK

**Giuseppe Moscelli**

UK

**David Mosen**

USA

**Mosa Moshabela**

South Africa

**Jin Mou**

Canada

**Joanna Moullin**

Australia

**Cheryl Moyer**

USA

**Maryam Mozooni**

Australia

**Ngatho Mugo**

Australia

**Ashar Muhammad Malik**

Pakistan

**Hilda A Mujuru**

Zimbabwe

**Elizabeta Mukaetova-Ladinska**

UK

**Kathleen Mulligan**

UK

**Ian Munabi**

Uganda

**Kenneth Munge**

Kenya

**Liubove Murauskiene**

Lithuania

**Moses Kinyanjui Muriithi**

Kenya

**Catriona Murphy**

Ireland

**Melanie Murray**

Canada

**Wilbroad Mutale**

Zambia

**Tshilidzi Muthivhi**

South Africa

**Ari Mwachofi**

USA

**Lucio Naccarella**

Australia

**Muhammad Naeem**

USA

**Somil Nagpal**

India

**Mkc Nair**

India

**Kent Nakamoto**

USA

**Miharu Nakanishi**

Japan

**Toshimi Nakanishi**

Japan

**Emmanuel Nakua**

Ghana

**Bertrand Nalpas**

France

**Susan Nancarrow**

Australia

**Annelies Nap**

Netherlands

**Corina Naughton**

UK

**Christopher Naugler**

Canada

**Justine Naylor**

Australia

**Jonathan Nebeker**

USA

**Edmund Nelson**

Hong Kong

**Susana Nery**

Australia

**Jenny Neuburger**

UK

**Cory Neudorf**

Canada

**Heather Neuman**

USA

**Louisa Ng**

Australia

**Nawi Ng**

Sweden

**Thinh Nguyen**

Australia

**Roch Nianogo**

USA

**Michael Nicholl**

Australia

**Davide Nicolini**

UK

**Masakazu Nishigaki**

Japan

**Godswill Nnaji**

Nigeria

**Natasha Noble**

Australia

**Ellen Nolte**

UK

**Declan Noone**

Ireland

**Ross Norman**

Canada

**Nicola North**

New Zealand

**Robyn Norton**

Australia

**Elisabetta Notarnicola**

Italy

**Mary Patricia Nowalk**

USA

**Carla Nunes**

Portugal

**Sabina Nuti**

Italy

**Monica Nyström**

Sweden

**Francis Obare**

Kenya

**Angela Obasi**

UK

**Konrad Obermann**

Germany

**Eivor Oborn**

UK

**Beverley O'Brien**

Canada

**Carol Obure**

Switzerland

**Alicia O'Cathain**

UK

**Jessica Ochalek**

USA

**Paul O'Connor**

Ireland

**Owen O'Donnell**

Greece

**Katsuhiko Ogasawara**

Japan

**James Ogilvie**

USA

**Adesola Ogunniyi**

Nigeria

**Ekechi Okereke**

Nigeria

**Jiro Okochi**

Japan

**Chibugo Okoli**

Nigeria

**Juan Oliva-Moreno**

Spain

**Vinicius Cunha Oliveira**

Brazil

**Kate O'Loughlin**

Australia

**Rose Mari Olsen**

Norway

**Lucy O'Malley**

UK

**Azizah Omar**

Malaysia

**Tracy Onega**

USA

**Mei-Sing Ong**

Australia

**Pierre Ongolo-Zogo**

Cameroon

**Chima Onoka**

Nigeria

**Joseph Orange**

Canada

**Pedro Ordunez**

USA

**Claire O'Reilly**

Australia

**Aaron Orkin**

Canada

**Lise Rosendal Østergaard**

Denmark

**Laysha Ostrow**

USA

**Helen O'Sullivan**

UK

**Songthip Ounpraseuth**

USA

**Kevin Outterson**

USA

**Shirley Owusu-Ofori**

Ghana

**Jan Oyebode**

UK

**Koyejo Oyerinde**

USA

**Lauren Pacek**

USA

**Timothy Page**

USA

**Ligia Paina**

USA

**Lubna Pal**

USA

**Alvisa Palese**

Italy

**Penny Paliadelis**

Australia

**Samiran Panda**

India

**Jaideep Pandit**

UK

**Massimiliano Panella**

Italy

**Grace Pang**

Singapore

**Supasit Pannarunothai**

Thailand

**Athanasia Papazafiropoulou**

Greece

**Yin Paradies**

Australia

**Guillermo Paraje**

Chile

**Jae Sung Park**

South Korea

**Young-Taek Park**

South Korea

**Seo Young Park**

USA

**Louise Parker**

USA

**Sarah Parker**

USA

**Justin Parkhurst**

UK

**Divya Parmar**

UK

**Carlos Parro-Torres**

Spain

**Gareth Parry**

USA

**Sonak Pastakia**

Kenya

**Tejal Patel**

Canada

**Preeti Patel**

UK

**Mitesh Patel**

USA

**Minal Patel**

USA

**Andrea Patey**

Canada

**Elisabetta Patorno**

USA

**Tilleul Patrick**

France

**Elizabeth Patterson**

Australia

**Mike Paulden**

Canada

**Julie Pavlin**

USA

**Ioanna Pavlopoulou**

Greece

**Milena Pavlova**

Netherlands

**Shelley Peacock**

Canada

**Christopher Pearce**

Australia

**Sallie Pearson**

Australia

**Nasheeta Peer**

South Africa

**Susan Peirce**

UK

**Salvador Peiro**

Spain

**Blanca Pelcastre**

Mexico

**Mikko Peltola**

Finland

**Marco Pereira**

Portugal

**Julian Perelman**

Portugal

**Ricardo Perez-Cuevas**

Mexico

**Lin Perry**

Australia

**Helen Perry**

USA

**Josefine Persson**

Sweden

**Vivekanandan Perumal**

India

**Michele Peters**

UK

**Stefan Peterson**

Sweden

**Goran Petersson**

Sweden

**Varduhi Petrosyan**

Armenia

**Christian Pettker**

USA

**Constanze Pfeiffer**

Switzerland

**Nittaya Phanuphak**

Thailand

**Ciaran Phibbs**

USA

**Craig Phillips**

Canada

**James Phillips**

USA

**Steven Phillips**

USA

**Gretchen Piatt**

USA

**Raynald Pineault**

Canada

**Luigi Pinnarelli**

Italy

**Amy Pinsent**

UK

**Jorge Pinzon**

Canada

**Vincent Piriou**

France

**Stephen Pitts**

USA

**Elena Platonova**

USA

**Virginia Plummer**

Australia

**Monika Pogorzelska-Maziarz**

USA

**Eng Hong Pok**

Malaysia

**Subhash Pokhrel**

UK

**Julie Polisena**

Canada

**Mary Politi**

USA

**Antonius J Poot**

Netherlands

**Ioana Popovici**

USA

**Mark Porcheret**

UK

**Filipe Portela**

Portugal

**Douwe Postmus**

Netherlands

**Iryna Postolovska**

Ukraine

**Malcolm Potts**

USA

**Mikaela Poulymenopoulou**

Greece

**Emma Power**

Australia

**James Powes**

USA

**Alexis Pozen**

USA

**Kay Price**

Australia

**Ignacio Prieto-Egido**

Spain

**Ari Probandari**

Indonesia

**Janice Probst**

USA

**Camelia Protopopescu**

France

**Janet Prvu Bettger**

USA

**Eve Puffer**

USA

**Thandi Puoane**

South Africa

**Sarah Purdy**

UK

**Mary Ellen Purkis**

Canada

**Jonathan Purtle**

USA

**David Putrino**

USA

**Tarik Qassem**

UK

**Feng Qian**

USA

**Fernanda Queirós**

Brazil

**Tom Quinn**

UK

**Fauziah Rabbani**

Pakistan

**Helena Rabie**

South Africa

**Borsika A Rabin**

USA

**Valeria Rac**

Canada

**Jany Rademakers**

Netherlands

**Kavita Radhakrishnan**

USA

**Whitney Raglin Bignall**

USA

**Momotazur Rahman**

USA

**Rahbel Rahman**

USA

**Maria Raikou**

UK

**Gururaghavendran Rajesh**

India

**Vahid Rakhshan**

Iran

**Anna Ralph**

Australia

**Bram Ramaekers**

Netherlands

**Kaushik Ramaiya**

Tanzania

**Helena Ramalhinho**

Spain

**Pilar Ramirez-Garcia**

Canada

**Lebogang Ramma**

South Africa

**Tao Ran**

USA

**Isuru Ranasinghe**

Australia

**Sue Randall**

Australia

**Padmaja Rani**

India

**Nicole Rankin**

Australia

**Ajaykumar Rao**

USA

**Rajah Rasiah**

Malaysia

**Garth Rauscher**

USA

**Joanna Raven**

UK

**Sundari Ravindran**

India

**Jennifer Raymond**

USA

**Mohammad Habib Raza**

India

**Tom Reader**

UK

**Jennifer Reath**

Australia

**Bernd Rechel**

UK

**Marcus Redley**

UK

**Gareth Rees**

New Zealand

**John Reeves**

Australia

**Oliver Reich**

Switzerland

**Glen Reid**

Australia

**Colin Reid**

Canada

**Stephen Reid**

South Africa

**Marcus Reidenberg**

USA

**Sharon Reif**

USA

**Lennart Reifels**

Australia

**Jan D. Reinhardt**

China

**Cristina Renzi**

UK

**Joseph Restuccia**

USA

**Valesca Retel**

Netherlands

**Andrea Reupert**

Australia

**Joel Rhee**

Australia

**Rebecca Richards-Kortum**

USA

**Julie Richardson**

USA

**Nathaniel Rickles**

USA

**Debra Rickwood**

Australia

**Traci Rieckmann**

USA

**Susan Rifkin**

UK

**Michael Rigby**

Ireland

**Iiris Riippa**

Finland

**William Riley**

USA

**Grant Ritter**

USA

**Philip Ritter**

USA

**Bjarne Robberstad**

Norway

**Mark Robbins**

USA

**Glenn Robert**

UK

**Adam Roberts**

UK

**Jane Robertson**

Australia

**James Robinson**

USA

**Dylan Roby**

USA

**Paul Robyn**

USA

**Rashmi Josephine Rodrigues**

India

**Rosa Rodriguez-Monguio**

USA

**Braeden Rogers**

USA

**Nikki Rogers**

USA

**Carlos Rojas-Fernandez**

Canada

**Anne Marie Mork Rokstad**

Norway

**Maria Francesca Romano**

Italy

**John Romley**

USA

**Claire Rondet**

France

**Janetta Roos**

South Africa

**Aldo Rosano**

Italy

**Michael Rosato**

UK

**Danielle Rose**

USA

**Pauline Rosenau**

USA

**Glenn Rosenbluth**

USA

**Meagen Rosenthal**

USA

**Andrew Rosenthal**

UK

**Michelle Rotermann**

Canada

**Thomas Rotter**

Canada

**Juan Ruano**

Spain

**Peter Ruesch**

Switzerland

**Carolyne Ruhembe**

Tanzania

**Corrine Ruktanonchai**

USA

**Simon Rule**

UK

**Tom Russ**

UK

**Elizeus Rutebemberwa**

Uganda

**Matthew Rutman**

USA

**David Ryan**

Canada

**Julia Ryan**

USA

**Sabe Sabesan**

Australia

**Ramon Sabes-Figuera**

Spain

**Mohsen Sadatsafavi**

Canada

**Cheryl Sadowski**

Canada

**Chandan Saha**

USA

**Jyotiranjan Sahoo**

India

**Rehana Salam**

Pakistan

**Sandra Saldivia**

Chile

**Gregory Salinas**

USA

**Stacie Salsbury**

USA

**Amber Salter**

USA

**Thelma Samuels**

Jamaica

**Caroline Sanders**

UK

**Felipe Sandoval**

Japan

**Silvina Santana**

Portugal

**Andrew Sarkin**

USA

**Pooria Sarrami Foroushani**

Australia

**Benn Sartorius**

South Africa

**Mikiya Sato**

Japan

**Yothin Sawangdee**

Thailand

**Peter Scanlon**

UK

**Frederieke Schaafsma**

Australia

**Bernhard Schaller**

UK

**Renee Scheepers**

Netherlands

**Mary Ann Scheirer**

USA

**Lori Schindel Martin**

Canada

**Virginia Schmied**

Australia

**Carl Schneider**

Australia

**Helen Schneider**

South Africa

**Kathryn Schnippel**

South Africa

**Martin Schoen**

USA

**Stephen Schoenbaum**

USA

**Adrian Schoo**

Australia

**Miranda Schram**

Netherlands

**Joseph Schulman**

USA

**Dena Schulman-Green**

USA

**Tim Schultz**

Australia

**Ellen Schultz**

USA

**Richard Schulz**

USA

**Linnaea Schuttner**

USA

**Nadine Schuurman**

Canada

**Rene Schwendimann**

Switzerland

**Anthony Scott**

Australia

**David Seal**

USA

**David Seder**

USA

**Janet Seeley**

Uganda

**Richard Segal**

USA

**Chiara Seghieri**

Italy

**Nicolas Senn**

Switzerland

**Tetine Sentell**

USA

**Hsien Seow**

Canada

**Albert Sese**

Spain

**Christopher Seymour**

USA

**Aviv Shachak**

Canada

**Asrul Shafie**

Malaysia

**Asm Shahabuddin**

Bangladesh

**Vandad Sharifi**

Iran

**Bharati Sharma**

India

**Urvashi Sharma**

UK

**Gulshan Sharma**

USA

**Dominick Shaw**

UK

**Rod Sheaff**

UK

**Aali Sheen**

UK

**Edlira Shehu**

Germany

**Chan Shen**

USA

**Donald Shepard**

USA

**Diana Sherifali**

Canada

**Qian Shi**

USA

**Rahul Shidhaye**

India

**Euichul Shin**

South Korea

**Eva Shipp**

USA

**Takeru Shiroiwa**

Japan

**Olayinka Shiyanbola**

USA

**Kah-Ling Sia**

Australia

**Shannon Sibbald**

Canada

**Estelle Monique Sidze**

Kenya

**Mark Siedner**

USA

**Sheetal Silal**

South Africa

**Robert Silverman**

USA

**Daniela Sime**

UK

**Eleanor Singer**

USA

**Christine Siokou**

Australia

**Eduardo Siqueira**

USA

**Steve Sizmur**

UK

**Rita Sjöström**

Sweden

**Susan Skledar**

USA

**Nancy L Sloan**

USA

**Berglind Smaradottir**

Norway

**Suzanne Smeltzer**

USA

**Kirsten Smillie**

Canada

**Mitchell Smith**

Australia

**Timothy Smith**

Australia

**Sheree Smith**

Australia

**Catherine Smith**

USA

**Susan Smith**

Ireland

**Samuel Smith**

UK

**Kimberley Smith**

UK

**Katherine Smith**

UK

**Justin D. Smith**

USA

**Janet Smylie**

Canada

**Margie Snyder**

USA

**Natasha Sobers-Grannum**

Barbados

**Seidu Sofo**

USA

**Mohammad Reza Sohrabi**

Iran

**David Sommerfeld**

USA

**Hummy Song**

USA

**Atsushi Sorita**

USA

**Michelle Sotero**

USA

**Aurelia Souares**

Germany

**Olaitan Soyannwo**

Nigeria

**Kaan Sozmen**

Turkey

**Heiko Spallek**

USA

**Mark Spigt**

Netherlands

**Karen Spilsbury**

UK

**Magi Sque**

UK

**Allison Squires**

USA

**Tanja Srebotnjak**

USA

**Chandrashekhar Sreeramareddy**

Nepal

**Sanjeev Sridharan**

Canada

**Sarah Ssali**

Uganda

**Catherine Staes**

USA

**Martha Starr**

USA

**Jane Stein-Parbury**

Australia

**Tetiana Stepurko**

Ukraine

**Lotte Steuten**

Netherlands

**Stephanie Stock**

Germany

**Johannes Stoelwinder**

Australia

**Tim Stokes**

New Zealand

**William Stones**

UK

**Sabine Stordeur**

Belgium

**Marianne Storm**

Norway

**William Story**

USA

**Slavi Stoyanov**

Netherlands

**Lahn Straney**

Australia

**Shiela Strauss**

USA

**Maryann Street**

Australia

**Heather Stuckey**

USA

**Matej Stuhec**

Slovenia

**Christian Peter Subbe**

UK

**Sujha Subramanian**

USA

**Madhuri Sudan**

USA

**Takehiro Sugiyama**

Japan

**Mark Sujan**

UK

**Sheena Sullivan**

Australia

**Trudy Sullivan**

New Zealand

**Sarah Sullivan**

UK

**Mei Sun**

China

**Qiang Sun**

China

**Bruce Sunderland**

Australia

**Kurt Svardsudd**

Sweden

**Michael Swanoski**

USA

**Katherine Sward**

USA

**Kevin Sykes**

USA

**Margie Synder**

USA

**Sarah Taber**

Canada

**Breena Taira**

USA

**Hannah Tait Neufeld**

Canada

**Tadashi Takeshima**

Japan

**Juan Tamargo**

Spain

**Giorgio Tamburlini**

Italy

**Tara Tancred**

UK

**Ryan Tandjung**

Switzerland

**Ravi Tandon**

India

**Shenglan Tang**

USA

**Shinichi Tanihara**

Japan

**Judith Tanner**

UK

**Peter Tanuseputro**

Canada

**Hong Tao**

USA

**Edith A.M. Tarimo**

Tanzania

**Colman Taylor**

Australia

**Natalie Taylor**

Australia

**Myra Taylor**

South Africa

**Sandra Taylor**

USA

**Tim Tenbensel**

New Zealand

**Javier Terol Fernández**

Spain

**Angela Testi**

Italy

**Kriti Thapa**

USA

**Kednapa Thavorn**

Canada

**Nawanan Theera-Ampornpunt**

Thailand

**Geoffrey Thiele**

USA

**Harsha Thirumurthy**

USA

**Jill Thistlethwaite**

Australia

**Aliki Thomas**

Canada

**Jessica Thomas**

Denmark

**Marie Thomas**

UK

**Anne Thomas**

USA

**Caroline Thompson**

USA

**Sarah Thomsen**

Sweden

**Joanna Thorn**

UK

**Judith Thornton**

UK

**Einar Thorsteinsson**

Australia

**James Tibenderana**

Uganda

**Stephanie Tierney**

UK

**Maike Tietschert**

Netherlands

**Trond Tjerbo**

Norway

**Adam Todd**

UK

**Iwona Tomaszewska**

Poland

**Sonila Tomini**

Netherlands

**Stephanie Topp**

Australia

**Amin Torabipour**

Iran

**Aleksandra Torbica**

Italy

**Sara Torres**

Canada

**Maria Torres**

USA

**Alberto Torres Cantero**

Spain

**Stacey Tovino**

USA

**Christine Toye**

Australia

**Heather Traino**

USA

**Francesco Tramonti**

Italy

**Joan Tranmer**

Canada

**Charlene Treanor**

Ireland

**Carla Treloar**

Australia

**Jill Trenholm**

Sweden

**Lyndal Trevena**

Australia

**Abhai Tripathi**

USA

**Lakkhina Troeung**

Australia

**Marie-Claude Trudel**

Canada

**Jeroen Trybou**

Belgium

**Yafang Tsai**

Taiwan

**Aster Tsegaye**

Ethiopia

**Bernice Tsoi**

Canada

**Joyce Tsoka-Gwegweni**

South Africa

**Heather Tubbs Cooley**

USA

**Anais Tuepker**

USA

**Alex Tulloch**

UK

**Carolyn Turvey**

USA

**Victoria Tutag Lehr**

USA

**Rajesh Tyagi**

Canada

**Namrata Uberoi**

USA

**Jane Umeano-Enemuoh**

Nigeria

**Martin Underwood**

UK

**Karla Unger-Saldaña**

Mexico

**Pablo Tarsicio Uribe-Leitz**

USA

**Robin Urquhart**

Canada

**Christine Urquhart**

UK

**Ruchan Uslu**

Turkey

**Sharon Utz**

USA

**Sakthivel Vaiyapuri**

UK

**Peter Van Bogaert**

Belgium

**Janet Van Cleave**

USA

**Elske Van Den Akker**

Netherlands

**Neeltje Van Den Berg**

Germany

**Michael Van Den Berg**

Netherlands

**Jef Van Den Ende**

Belgium

**Iris Van Der Heide**

Netherlands

**Renée Van Der Leeuw**

Netherlands

**Philip Van Der Wees**

Netherlands

**Christel Van Dijk**

Netherlands

**Thamar Van Esch**

Netherlands

**Evert Van Leeuwen**

Netherlands

**Tom Vandersteegen**

Belgium

**Kris Vanhaecht**

Belgium

**Gamze Varol Saraçoglu**

Turkey

**Ashwin Vasan**

UK

**Elizabeth Vasquez**

USA

**Sukumar Vellakkal**

India

**Peter Ventevogel**

Netherlands

**Norma Verdolini**

Italy

**Itziar Vergara**

Spain

**Montserrat Vergara Duarte**

Spain

**Kim Verhaegh**

Netherlands

**Stefano Villa**

Italy

**David Vivas-Consuelo**

Spain

**Bruce Vogel**

USA

**Olaf Von Dem Knesebeck**

Germany

**Kranti Vora**

USA

**Thomas Vreugdenburg**

Australia

**Victoria Anne Wade**

Australia

**Julia Wade**

UK

**Adrian Wagg**

Canada

**Anita Wagner**

USA

**Olive Wahoush**

Canada

**Tomoko Wakui**

Japan

**David Walk**

USA

**Emily Walkom**

Australia

**Lars Wallin**

Sweden

**Bronagh Walsh**

UK

**Julia Walsh**

USA

**Merrilyn Walton**

Australia

**Richard Wamai**

USA

**Neng Wan**

USA

**Thomas Wan**

USA

**Nick Wan**

USA

**Thomas Wan**

USA

**Kunjie Wang**

China

**Qing Wang**

China

**Jian Wang**

China

**Chi-Chuan Wang**

Taiwan

**Jye Wang**

Taiwan

**Fahui Wang**

USA

**Frederick Wang**

USA

**Zhen Wang**

USA

**Paresh Wankhade**

UK

**Kim Ward**

South Africa

**Emily Warren**

UK

**Shuichiro Watanabe**

Japan

**Heather Waterman**

UK

**Claire Watt**

Kenya

**France Weaver**

Switzerland

**Sallie J. Weaver**

USA

**William Weeks**

USA

**Madeleen Wegelin**

Netherlands

**Li Wei**

UK

**Xiaolin Wei**

China

**Paolina Weidinger**

Sweden

**Marianne Weiss**

USA

**Yi-Hao Weng**

Taiwan

**Nicole Werner**

USA

**Catrin Wessman**

Sweden

**Michael West**

UK

**Daan Westra**

Netherlands

**Anna Whelan**

Australia

**Maxine Whittaker**

Australia

**Jeff Whittle**

USA

**David Whyatt**

Australia

**Sophie Whyte**

UK

**Ellen Wiebe**

Canada

**Natasha Wiebe**

Canada

**Therese Wiegers**

Netherlands

**Lisa Susan Wieland**

USA

**Michael Wiese**

Australia

**Siri Wiig**

Norway

**Charles Wiles**

UK

**Anne Wilkinson**

Australia

**Chris Wilkinson**

Australia

**Evi Willemse**

Belgium

**Lauren Williams**

Australia

**John Williams**

UK

**Cameron Willis**

Canada

**Karen Willis**

Australia

**Andrew Wilson**

UK

**Reda Wilson**

USA

**Vivian Lee Wing-Yan**

Hong Kong

**Rudi Wisaksana**

Indonesia

**Torbjørn Wisløff**

Norway

**Julia Witt**

Canada

**Holly Witteman**

Canada

**Mallory Woiski**

Netherlands

**Mulu Abraha Woldegiorgis**

Australia

**Fredric Wolinsky**

USA

**Wendy Wong**

Hong Kong

**Carlos King Ho Wong**

Hong Kong

**Yut Lin Wong**

Malaysia

**Erica Wood**

Australia

**John Woolham**

UK

**Richard Wootton**

Norway

**Sally Wortley**

Australia

**Zhuochun Wu**

China

**David Bin-Chia Wu**

Taiwan

**Dongfeng Wu**

USA

**Joanne Wu**

USA

**Laura Wyatt**

USA

**Lesley Wye**

UK

**Klaske Wynia**

Netherlands

**Kaspar Wyss**

Switzerland

**Jing Xie**

USA

**Fenglian Xu**

Australia

**Judy Xu**

China

**Di Xue**

China

**Joanne Yaffe**

USA

**Ann Yamada**

USA

**Noriko Yamamoto**

Japan

**Thespina Yamanis**

USA

**Yui Yamaoka**

Japan

**Fei Yan**

China

**Li Yang**

China

**Yi-Hsin Yang**

Taiwan

**Tracey Yap**

USA

**Lucy Yardley**

UK

**Patsy Yates**

Australia

**Alfred Yawson**

Ghana

**Bahareh Yazdizadeh**

Iran

**Jan Ybema**

Netherlands

**Laurann Yen**

Australia

**Siyan Yi**

Cambodia

**Getnet Yimer**

UK

**Cheng-Har Yip**

Malaysia

**Linda Yoder**

USA

**Jean Yoon**

USA

**Marcel Yotebieng**

USA

**Doris Young**

Australia

**Tracey Young**

UK

**Mustafa Younis**

USA

**Susan Yount**

USA

**Ping Yu**

Australia

**Weiyu Yu**

China

**Zhi-Gang Yu**

China

**Xinhua Yu**

USA

**Wen Yufeng**

China

**Norsiah Yunus**

Malaysia

**Kazeem Yusuff**

Nigeria

**Nabila Zaka**

Thailand

**Margareth Zanchetta**

Canada

**Ehsan Zarei**

Iran

**Ruth Zaslansky**

Germany

**Jennifer Zelmer**

Canada

**Elisabeth Zemp Stutz**

Switzerland

**Jimmy Zeng**

New Zealand

**Wu Zeng**

USA

**Jianzhen Zhang**

Australia

**Puhong Zhang**

China

**Xinping Zhang**

China

**Lingling Zhang**

USA

**Mei Zhao**

USA

**Shuang Zhong**

China

**Zhongliang Zhou**

China

**Tracy Y Zhu**

Hong Kong

**Junya Zhu**

USA

**Diana Zidarov**

Canada

**Monica Zolezzi**

Qatar

**Anna Zorzet**

Sweden

**Beate Zschorlich**

Germany

**Sandra Zwijsen**

Netherlands

